# Low-cost, open-source device for applying controlled olfactory stimuli during functional magnetic resonance imaging (fMRI) of the brain

**DOI:** 10.1016/j.ohx.2025.e00708

**Published:** 2025-09-23

**Authors:** Raffaella Salama, Miguel A. Rodríguez-Lázaro, Camil Castelo-Branco, Iñigo Herrero-Vidaurre, Laura Ribera-Torres, Emma Muñoz-Moreno, Jorge Otero, Ramon Farré

**Affiliations:** a Unit of Biophysics and Bioengineering, School of Medicine and Health Sciences, University of Barcelona, Casanova 143, 08036 Barcelona, Spain; b Clinic Institute of Gynecology, Obstetrics and Neonatology, Faculty of Medicine-University of Barcelona, Hospital Clínic of Barcelona, Barcelona, Spain; c Institut Investigacions Biomediques August Pi Sunyer, Barcelona, Spain; d Magnetic Resonance Imaging Core Facility, IDIBAPS, Barcelona, Spain; e CIBER of Respiratory Diseases (CIBERES), Monforte de Lemos 3-5, 28029 Madrid, Spain

**Keywords:** Smell sense, Olfactometer, Olfactory pathway, Neural response, fMRI, Pheromone, Arduino control, Airflow sequence

## Abstract

Using functional magnetic resonance imaging (fMRI) to assess brain activity in response to olfactory stimuli is of great biomedical and clinical interest. However, application of controlled sequences of olfactory stimuli within the setting of fMRI equipment is challenging since the associated limitations for non-magnetic and non-conducive materials. Here, we have developed and tested a simple, low-cost, open-source, stand-alone device to apply selectable controlled sequences of olfactory stimuli in subjects undergoing fMRI. The device consists of an Arduino-controlled unit containing a blower-based airflow generator and a multichannel air valves system, a set of long air conducting tubing, and bubbler-based odor sources. The device was first validated on the bench to characterize the range of achievable flows, ensuring that the device can be adapted to a variety of applications and fMRI settings. The effectiveness of device performance was subsequently assessed in female patients with sexual dysfunction using a fMRI protocol based on subjecting them to controlled sequences of olfactive stimuli with pheromone, phenethyl alcohol (a rose fragrance) or neutral clean air. Therefore, the device facilitates biomedical research and clinical assessment of the neural pathways modulated by the olfactory system.

## Graphical abstract

**Figure:**
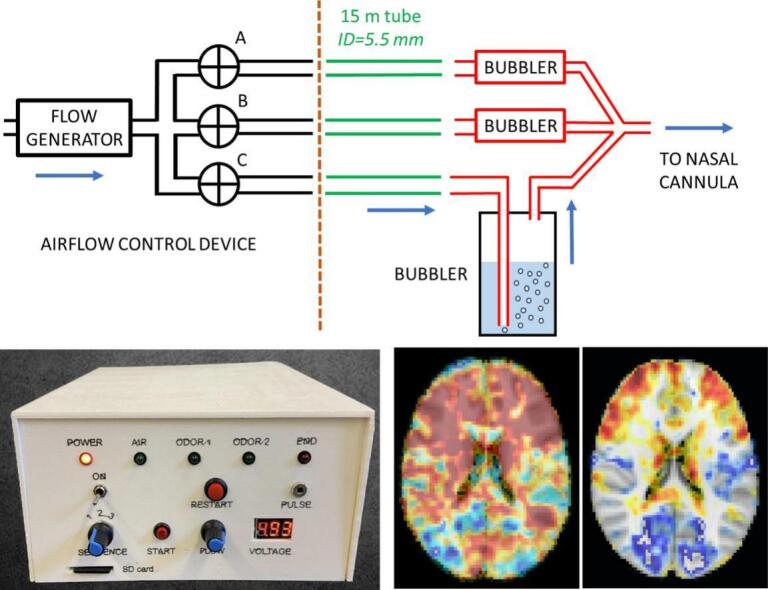

**Specifications table**

**Table:** 

Hardware name	Device for applying controlled olfactory stimuli during functional magnetic resonance brain imaging
Subject area	Medicine
Hardware type	Device for human biomedical research
Closest commercial analog	No commercial analog is available
Open source license	GPL v3
Cost of hardware	The total cost of the material for the device building is ≈ 250 € (as of June 2, 2025).
Source file repository	https://data.mendeley.com/datasets/g896nz5gm6/1 https://doi.org/10.17632/g896nz5gm6.1

## 1 Hardware in context

Functional magnetic resonance imaging (fMRI) is a relatively recent technique to map the activation of different brain areas and thus to track the neural response to specific external stimuli, including those elicited through the olfactory system [1,2]. Studying brain activation by consciously perceived (e.g. perfumes) or unperceived (e.g. pheromones) stimuli sensed through olfaction is of increasing socio-economical and medical interest. Indeed, although still poorly understood, we already know that olfactory stimuli may modulate interpersonal relationships [3,4] and consumer attitudes [5]. Very remarkably, the olfactive response is altered in different pathological conditions, for instance in the case of certain virus infections [6] and in a wide range of dysfunctions affecting the brain and neural system such as epilepsy [7], depression [8], anxiety [9], and Parkinson [10] and Alzheimer [11] diseases.

Widely extending fMRI applications to study the neural pathways in olfactory function has been partially hindered by the technical complexity of designing a device to apply controlled odors specifically to patients subjected to fMRI. Such a device requires that its components that are placed close to the patient, thus inside the MRI room, are made of non-magnetic and non-conductive materials. Moreover, it is required that the device components providing the sequence of odors applied to the patient, which are placed several distant meters outside the MRI room, perfectly controls the composition and time sequence of the air reaching the subject’s nostrils. Most devices available for odor stimulation are not suitable for fMRI [12] and the different settings that have been proposed for such a function are either complex, expensive, with slow response time or require an external pressurized air source [13,14,15]. As a result, there is very limited availability of devices for easily applying olfactory stimuli which make it difficult to carry out olfactory related fMRI studies.

To facilitate biomedical research on and clinical assessment of the neural pathways modulated by the olfactory system, we have developed and tested a simple, low-cost, open source, stand-alone device allowing to apply easily selectable controlled sequences of olfactory stimuli in subjects undergoing fMRI.

## 2 Hardware description

**Fig. 1:**
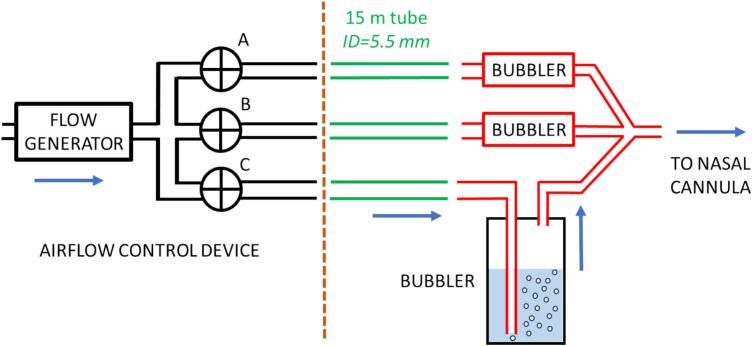
Diagram of the device. Note that only one of the 3 identical bubblers is shown in detail. The blue arrow represents the airflow when valve C is open. See text for explanation.

The optimized device described here consists of three different sections: 1) a control unit containing a blower-based airflow generator and a multichannel air valves system, 2) a set of long air conducting tubing, and 3) bubbler-based odor sources (Fig. 1). The components placed at the right of the brown vertical dashed line in Fig. 1 are made of non-magnetic, non-conductive materials to be placed inside the MRI room.

The blower-based flow generator takes room air, and its pressurized outlet is connected in parallel to the inlet of 3 electrovalves (A, B, C) which remain closed when not activated. Each valve outlet is connected to an air conducting non-collapsible plastic tube (5.5 mm ID) with a length of ≈15 m, sufficient to place the control components outside the MRI room (e.g., at the side of the control console of the fMRI system). At any time, the valve controller opens only one of the valves (A,B, or C) according to a selectable time sequence. The outlet of each conducting tube coming from valves A, B, and C is connected to a bubbler containing pure water (non-odor control) or solutions of the two odors to be tested. Each bubbler is made with a 100 ml glass bottle and the liquid height that the bubbles must circulate is ≈3 cm. The bubblers are placed close to the subject’s feet on the MRI patient table, with their outlets connected at the inlet of the subject’s nasal cannula (Fig. 1). It is important to note that control images should be acquired by using a pure air flow (instead of switching off the device) to avoid potential differences in brain response introduced by tactile flow perception.

As the three parallel airflow pathways connected to each valve outlet are identical (except for odor component dilution in each bubbler), they present the same airflow resistance and thus the airflow reaching the nasal prongs is the same for the three pathways. The airflow magnitude is determined by the blower speed (voltage-regulated). The voltage-airflow relationship depends on the dimensions of each setting (airflow resistances of the valve, tubing, bubbler, and nasal prongs) and thus should be determined for each facility. Given that the outlets of the three parallel pathways are connected close to the entrance of the nasal prongs, the mixing air volume is virtually nil and thus the switch of the odors reaching the subject’s nostrils is very fast.

Indeed, the time delay from valve switching to the new odor arriving to the patient’s nares depends on the airflow and the distance from the bubbler to the nares. If the bubblers were placed outside the fMRI room, this time could be considerably high. But in our setting the tubing from any bubbler and the patient’s nares is just the nasal cannula (Fig. 1). As conventional nasal cannulas (e.g. those for oxygen therapy) have an internal diameter of 4 mm, and considering a length of 2 m (enough for connecting the bubbler at the feet of the MRI bed to the patient’s nares), the dead space into the cannula is ≈25 ml. Given that the airflow is typically 2 l/min, overcoming the dead space takes ≈0.75 s, thus the change in gas odor has a very small delay.

The sequence of odors is triggered by a digital synchrony signal sent by the fMRI scanner when image acquisition starts.

## 3 Design files summary

All the design and software files (Table 1) necessary to build the device presented in this work are distributed under the GPL v3 license and they can be found in the supplementary materials of this manuscript at the following public repositories:

**Table 1:** Design file summary table.

**Design file name**	**File type**	**Open source license**	**Location of the file**
Enclosures and lids	STL	GPL v3	STL files folder
Code	ino file	GPL v3	Arduino Code folder
PCB Layout	pdf and jpg file	GPL v3	Electronics folder

https://data.mendeley.com/datasets/g896nz5gm6/1

https://doi.org/10.17632/g896nz5gm6.1

## 4 Bill of materials summary

The total cost of the materials (Table 2) for building the device is 251.91€ as of June, 2nd, 2025.

**Table 2:** Bill of materials summary table.

**Designator**	**Component**	**Quantity**	**Cost per unit €**	**Total cost Currency €**	**Source of materials**	**Material**
Electronic switch	Transistors TIP122	3	2,20	6,60	https://www.amazon.it/Transistor-Darlington-TIP122-Microelectronics-pezzi/	Non-specific
Electronic switch	Diodes 1N4007	3	0,04	0,12	https://www.amazon.es/AUKENIEN-rectificadores-voltios-silicio-electr%C3%B3nicos/	Non-specific
Resistor	Resistor	15	0,04	0,60	https://www.amazon.es/ALLECIN-Resistor-Pel%C3%ADcula-5-resistencias/	Non-specific
Capacitor	Capacitor	1	0,05	0,05	https://www.amazon.es/BOJACK-Condensadores-tipos-unidades-caja/	Non-specific
Indicator	Leds	5	0,02	0,10	https://www.amazon.es/AUKENIEN-Redondo-Difusos-Amarillo-50piezas/dp/B0972D2BMH/	Non-specific
Optocoupler	Optocoupler H11L	1	5,74	5,74	https://www.amazon.es/OPTOCOUPLER-Schmitt-Optoacopladores-Opto-Electr%C3%B3nica-Cantidad/	Non-specific
Plugs	Connectors					Metal
Microcontroller addon	SD module	1	5,49	5,49	https://www.amazon.es/azdelivery-Reader-Tarjeta-memoria-Arduino/	Non-specific
Mem card	SD card	1	9,99	9,99	https://www.amazon.es/SanDisk-High-Endurance-Tarjeta-videovigilancia/	Non-specific
Dimmer	Potentiometer	1	0,89	0,89	https://www.amazon.es/GTIWUNG-Potenci%C3%B3metro-Rotativo-Terminales-Potenciometro/	Non-specific
Display	LED display	1	5,99	5,99	https://www.amazon.es/CABLEPELADO-Pantalla-Precisi%C3%B3n-Compacto-Electr%C3%B3nicos/	Non-specific
Buttons	Push buttons	2	0,35	0,70	https://www.amazon.es/Runcci-Yun-Pulsadores-Electricos-Interruptor-Momentaneo/	Non-specific
Key	Switch	1	1,67	1,67	https://www.amazon.es/ARTGEAR-Conmutador-Interruptor-Posiciones-Vehiculo/	Non-specific
Valves	Solenoid valves	3	59,46	178,38	https://es.rs-online.com/web/p/valvulas-de-solenoide/	Non-specific
Microcontroller	Arduino Nano	1	3,80	3,80	https://www.amazon.es/APKLVSR-Module-mega328P-Arduino-Puerto/	Non-specific
Box	3D printer reel	523 g	30€	30€		Polymer

## 5 Build instructions

### 5.1 3D design and printing

**Fig. 2:**
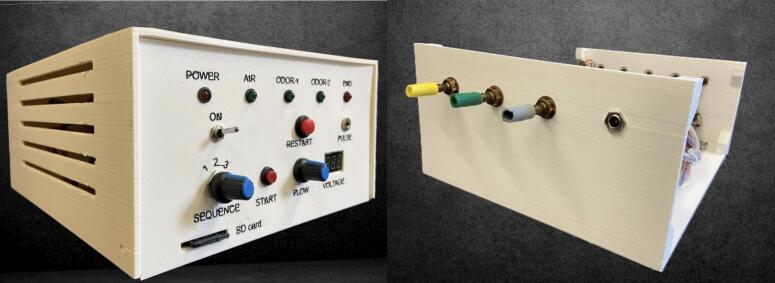
Front and rear view of the Control unit.

The electronic control system is housed within a custom-designed 3D-printed enclosure, created using Fusion 360 software (Fig. 2). The enclosure, shaped as a rectangular parallelepiped measuring 24x20x10 cm^3^, consists of a main body and a detachable lid secured together with screws to ensure both structural integrity and convenient access to internal components.

**Fig. 3:**
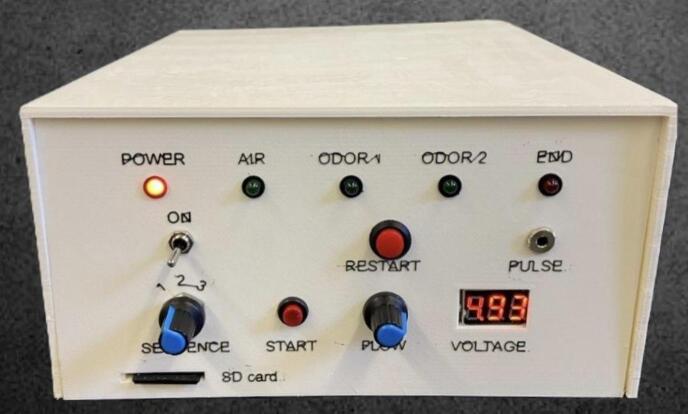
Detail of the front panel of the control unit (see text for explanation).

The front panel (Fig. 3) features a comprehensive user interface for system control and monitoring, incorporating five status LEDs that provide visual feedback on system power, active odor channels, active airflow, and an “end” indicator that signals the completion of the odor delivery sequence when no valves are active. Additionally, it includes a power switch to control the main power supply and a restart button that opens all three valves for 12 s to purge the system. A rotary commutator enables the selection of one of the desired odor-sequence files stored on an SD card, while a start button initiates the selected sequence immediately after receiving a synchronization pulse, allowing the device to synchronize with external signals coming from the fMRI machine. The front panel also includes an easily accessible SD card slot for updating or modifying the stored sequences, a potentiometer that allows manual control over the voltage supplied to the blower to regulate airflow, and an LED display that provides real-time feedback on the airflow setting by showing the applied voltage. The rear panel (Fig. 2) is equipped with dedicated air connectors for the three solenoid valves and the power supply. To ensure effective thermal management, the side panels are fitted with strategically placed ventilation slots, promoting airflow circulation and preventing overheating of internal components.

### 5.2 Electronics

**Fig. 4:**
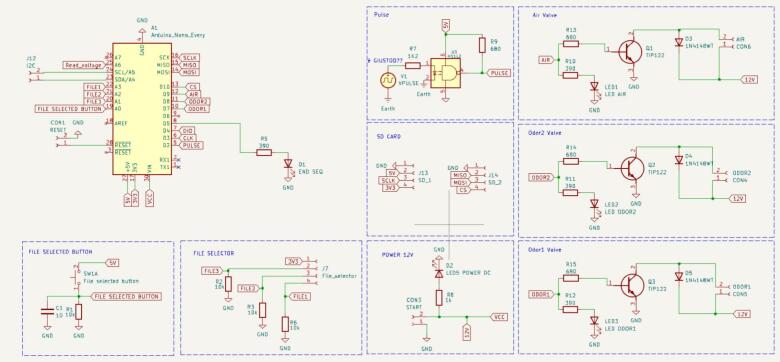
Schematic designed in KiCad.

**Fig. 5:**
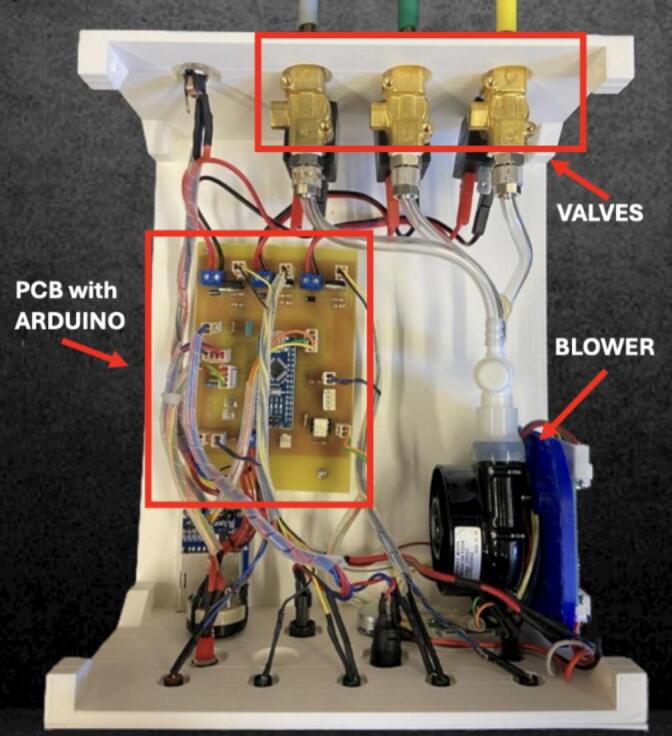
Final set-up.

The schematic of the implemented electronic circuit is shown in Fig. 4 and, along with the printed circuit board (PCB), was designed by using KiCad software. The final set-up is shown in Fig. 5. The schematic outlines a compact electronic control system base on an Arduino Nano microcontroller. The system is designed to automate the activation of three solenoid electro-valves – two for odor delivery and one for room airflow – based on operator-selected sequences. It features a pulse generation unit to synchronize timing events, an SD card interface for reading pre-programmed odor sequences, and the user interface components, including a rotary file selector switch and a confirmation button to start the selected sequence.

The system is powered by a 12 V DC external supply source. The circuit comprises three valve driver blocks, each controlling an L172 Series 2-port solenoid valve—direct-acting electromagnetic valves designed for airflow applications. Each driver employs a TIP122 NPN Darlington transistor, which acts as a switch controlled by the digital signals (AIR, ODOR1, or ODOR2) sent from the microcontroller, thereby opening the valve and enabling airflow. A flyback diode is placed in parallel with each solenoid coil to protect the transistor from voltage spikes caused by the inductive nature of the coil during switching.

A LED indicator allows the operator to see which valve is opened and therefore which scent is being released to the subject. The valves are connected to a 12 V high-pressure blower that regulates airflow. The speed of the blower can be adjusted by the operator through a potentiometer.

The system includes an SD card module, connected to Arduino via the SPI, which stores 3 files containing predefined odor delivery sequences. Each file specifies which valves to activate and for how long, allowing flexible control without modifying the firmware. The module operates at a 3.3 V logic level. File selection is done using a rotary commutator, externally mounted, which allows the operator to choose among the three preset sequences by sending a digital signal to the Arduino through dedicated input lines. A confirmation button triggers – when pressed – the microcontroller to start executing the odor sequence selected by the operator from the SD card.

### 5.3 Arduino control

The embedded system implemented is designed to control odor stimulation through solenoid valves, as well as to execute predefined instructions stored on an SD card. The firmware handles the entire process, including system initialization, SD-based instruction execution, and valve control. At the beginning, the system initializes the SPI interface by setting the baud rate and defining the pins used for communication with the SD card. At power-on, the system automatically runs a cleaning procedure, opening all valves for 12 s to purge the system, ensuring that the chamber is cleared before starting any odor delivery sequence. After the purge, all valves are closed, and the system waits for the operator input signal. The system continuously monitors the position of the commutator, connected to the digital input pins. These inputs determine which file of odor-sequence is selected for execution. When the operator presses the selection button, the system reads the corresponding sequence file stored on the SD card and begins its execution.

Each sequence file is a. txt file and each line of the file contains the name of the valve and the corresponding duration time (in seconds) for which the valve should remain open (e.g., odor1, 10). See file example in Supplementary material. The system reads the file line by line and executes the actions sequentially. Once a sequence begins, it cannot be interrupted or modified; the firmware only checks the commutator’s position again after the current sequence has completed; at the end of the sequence, an “end” LED is turned on, providing a clear visual signal to the operator that the sequence has finished.

## 6 Operation instructions

The device has been designed to facilitate its adaptation to the setting of each fMRI application site. The section and length of the conducting tubes (e.g. 15 m) can be increased or reduced as required (but maintained of the same length for all the tubes used to avoid differences in the resistance of the tubes that will result in different flows for the different lines), the bubbling units can be constructed using the simplest available recipients, and any nasal cannula for clinical application can be used (e.g., the ones for non-invasive oxygen therapy). Once the tubing-bubbler-nasal prongs assembly is established for a specific fMRI setting, the only variable to adjust is the voltage regulating the blower to achieve the desired airflow magnitude. To this end, three possible procedures can be followed. The simplest but less precise one is by adjusting the airflow without measuring it, just by subjectively sensing the amount of airflow at the nostrils by experienced staff. However, it is recommended to measure actual airflow for the sake of standardization of odor stimuli intensity. In the case of using a flowmeter for setting the blower driving voltage, it is important to ensure that its airflow resistance is negligible as compared with the resistance of the whole air pathway, otherwise the measured flow would be different from the one arriving to the patient’s nostrils when the flowmeter is not present. Interestingly, the most simple and accurate option for measuring the airflow is to proceed with the following steps: 1) connecting the outlet of the nasal prongs to an empty soft-wall bag (impedance to air flow virtually nil), 2) allowing airflow into the bag for a given time (Δt) (avoiding bag wall tension), 3) closing and disconnecting the bag, 4) measuring the gas volume (ΔV) by removing it with a conventional syringe, and 5) computing flow as ΔV/Δt.

To ensure that the airflow is the same for the three parallel pathways, it is important to ensure that the height of water that the air bubbles must travel inside the bubbler (Fig. 1) is the same in all bubblers. It should also be noted that the three parallel air pathways must be connected (even in case of testing only one odor and air control). Otherwise, most airflow from any other lines would leak into the room thus would not reach the nasal prongs and the patient.

The operator can define any sequence of odors application (i.e., of valves opening) since the device includes three pre-loaded on–off valve sequences in a memory card. The operator can easily define any odor time sequence by simply modifying the patterns stored in the files card.

The setting described here is designed for using two odorants and one room air control (3 bubblers). However, 5 more additional parallel lines could be easily added, each with different odorant concentrations or with different odorants in the bubblers. From the control viewpoint, such a modification would only require adding more electrovalves (and on/off led displays, not compulsory) and to rewrite a few lines in the Arduino code. As they are in parallel, adding odor lines would not affect the performance of the blower-based flow generation.

As the long tubes (in green in Fig. 1) experience only unidirectional flow of fresh room air, they cannot be contaminated by odors and thus they should not be replaced regularly. However, to avoid potential odor contamination, changing the type of odor in the setting requires to replace the bubbler and its immediately connecting tubes (in red in Fig. 1). The nasal cannula is for individual use and thus is replaced for each patient.

At the end of each measurement in a patient, when the used nasal cannula is discarded, the outlet from the bubblers should be occluded until a new cannula for another patient is connected. This will avoid passive diffusion of the highly volatile components from the bubblers to the nasal cannula and then to room air. As passive diffusion of the odors from each bubbler to the rest of the circuit can occur even in case the valves and the connection to the cannula are closed, it is recommended to disconnect the bubblers from the device when measurements are not being carried out.

The device described here, similarly to all the ones delivering odors to patients by a nasal cannula (recovering and expelling expired air would be cumbersome), runs a certain risk of volatile gas components contamination into the room, although the effect of staff or previous patients using parfums or other cosmetic products could be greater. Therefore, the MRI room should be particularly well ventilated when performing measurements on odor response.

## 7 Validation and characterization

The device we built as described here was validated first in the bench and subsequently in a clinical application. We characterized the range of airflow that can be achieved at the subject’s nostrils. Specifically, for the described setting, the flow at the nasal cannula can be progressively adjusted up to 2.7 l/min (for the maximum 5.0 V regulating voltage). At this point, the pressure generated at the entrance of the electrovalves (measured with a transducer not shown in Fig. 1 and not included in the device) was 23.4 hPa, thus the airflow resistance of the whole circuit was 8.7 hPa·s/ml. By using a pneumotachograph we confirmed that, for any given blower control voltage, the flow at the nasal cannula was virtually the same for the three parallel odor lines (differences within 1.2 %). Accordingly, the setting was able to generate the typical 2 l/min flow used in odor applications [16]. However, it is interesting to note that reducing the 15 m length of the tubing or slightly increasing its diameter would reduce the circuit airflow resistance and thus increase the flow generated. An alternative way to increase the flow for a given airflow resistance could be to increase the voltage supplying the blower from the current 12 V up to its nominal 24 V (this would require circuit modifications to keep the 12 V supply to the rest of the device components).

It should be mentioned that the technical validation described herein corresponds to the specific prototype device we built. Using components different from (although similar to) the ones described here may require technical adjustments or adaptations and therefore the final performance of the resulting device must be specifically assessed.

**Fig. 6:**
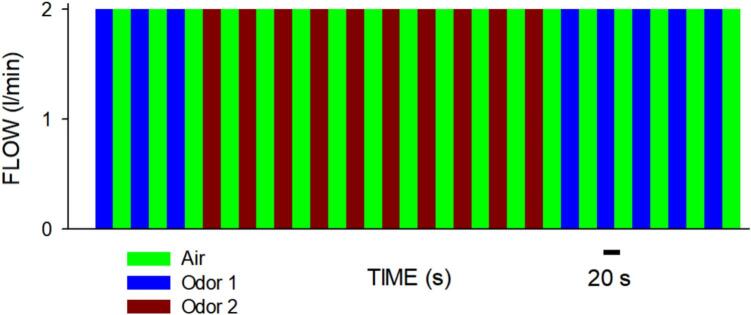
Example of a sequence of airflow with odors applied at the nasal cannula.

Fig. 6 shows an example of the odors sequence that can be applied. Each color indicates which of the valves was open (each 20 s) and thus which of the odors (or control air) was applied to the nostrils. As already mentioned, the device is triggered by a digital output from the MRI console to ensure synchronization between the start of the MRI image acquisition and the start of the olfactory stimulation. Once the protocol starts, every odor is applied for a period of 20 s. Considering that in adults the normal spontaneous breathing frequency is about 12 to 20 breaths/min (i.e. 3–5 s cycling period), it is ensured that the subject inhales the odor several times within the 20 s period, with only a slight variation in the first inhalation time. Since the processing takes into account the activation during the whole 20 s of odor stimulus to evaluate brain activation, a small variation in time of first inhalation has a negligible effect on the estimation of the response to the stimulus.

**Fig. 7:**
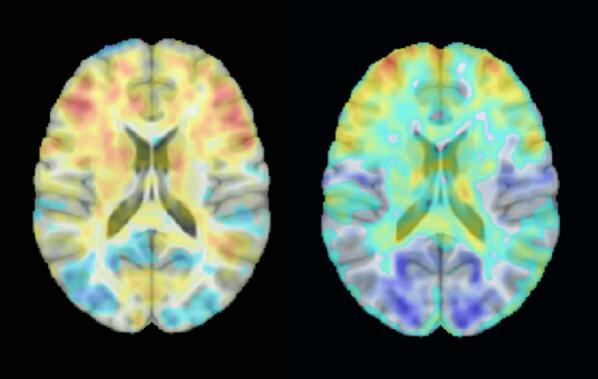
Mean activation map of 15 female patients subjected to olfactory stimuli with pheromone and rose odor, in both cases relative to control air (no olfactive stimulus). Red and blue correspond to areas of increased and decreased activation, respectively.

We tested the effectiveness of device performance in female patients included in a clinical study of sexual dysfunction. The fMRI protocol followed a standard block design in which participants were exposed to alternating sequences of air with scent of pheromone or phenethyl alcohol (a rose fragrance), or clean air (control). As an example, Fig. 7 shows the mean (15 patients) brain activation map in response to pheromone and rose odor stimuli, demonstrating a similar pattern of activation in areas related to olfaction, but with some differences in more occipital regions.

## Ethics statements

The clinical study applying olfactory stimuli during fRMI in patients was approved by the Ethical Board of Hospital Clínic (V1-27/03/2020). The study has been registered in Europe with an EudraCT code N° 2020-001514-38 and in the US clinical trials register with the code NCT04677491.

## CRediT authorship contribution statement

**Raffaella Salama:** Writing – review & editing, Writing – original draft, Validation. **Miguel A. Rodríguez-Lázaro:** Validation, Methodology. **Camil Castelo-Branco:** Validation, Conceptualization. **Iñigo Herrero-Vidaurre:** Validation, Conceptualization. **Laura Ribera-Torres:** Validation, Conceptualization. **Emma Muñoz-Moreno:** Validation, Conceptualization. **Jorge Otero:** Writing – review & editing, Methodology, Conceptualization. **Ramon Farré:** Writing – review & editing, Writing – original draft, Validation, Supervision, Conceptualization.

## Declaration of competing interest

The authors declare that they have no known competing financial interests or personal relationships that could have appeared to influence the work reported in this paper.
